# Evaluating the effects of S-ketamine on postoperative delirium in elderly patients following total hip or knee arthroplasty under intraspinal anesthesia: a single-center randomized, double-blind, placebo-controlled, pragmatic study protocol

**DOI:** 10.3389/fnagi.2023.1298661

**Published:** 2023-11-30

**Authors:** Youzhuang Zhu, Wei Feng, Qinghan Kong, Fang Sheng, Zhichao Li, Weilong Xu, Qun Li, Yan Han, Xiuyun Wu, Changxin Jia, Jie Guo, Yang Zhao

**Affiliations:** ^1^Department of Anesthesiology, The Affiliated Hospital of Qingdao University, Qingdao, Shandong Province, China; ^2^Department of Anesthesiology, Cancer Hospital Chinese Academy of Medical Science, Beijing, China; ^3^Department of Anesthesiology, Shandong Cancer Hospital and Institute, Shandong First Medical University and Shandong Academy of Medical Sciences, Jinan, Shandong, China

**Keywords:** S-ketamine, postoperative delirium, neurocognitive disorders, neuroprotective effect, pragmatic study

## Abstract

**Introduction:**

Postoperative delirium (POD) is an acute, transient brain disorder associated with decreased postoperative quality of life, dementia, neurocognitive changes, and mortality. A small number of trials have explored the role of S-ketamine in the treatment of POD due to its neuroprotective effects. Surprisingly, these trials have failed to yield supportive results. However, heterogeneity in delirium assessment methodologies, sample sizes, and outcome settings as well as deficiencies in S-ketamine use methods make the evidence provided by these studies less persuasive. Given the severe impact of POD on the health of elderly patients and the potential for S-ketamine to prevent it, we believe that designing a large sample size, and rigorous randomized controlled trial for further evaluation is necessary.

**Methods:**

This is a single-center, randomized, double-blind, placebo-controlled, pragmatic study. Subjects undergoing total hip or knee arthroplasty will be randomized in a 1:1 ratio to intervention (*n* = 186) and placebo (*n* = 186) groups. This trial aims to explore the potential role of S-ketamine in the prevention of POD. Its primary outcome is the incidence of POD within 3 postoperative days. Secondary outcomes include the number of POD episodes, the onset and duration of POD, the severity and subtype of POD, pain scores and opioid consumption, sleep quality, clinical outcomes, and safety outcomes.

**Discussion:**

To our knowledge, this is the first pragmatic study that proposes to use S-ketamine to prevent POD. We reviewed a large body of literature to identify potential preoperative confounding variables that may bias associations between the intervention and primary outcome. We will use advanced statistical methods to correct potential confounding variables, improving the test’s power and external validity of test results. Of note, the patient population included in this trial will undergo intraspinal anesthesia. Although large, multicenter, randomized controlled studies have found no considerable difference in the effects of regional and general anesthesia on POD, patients receiving intraspinal anesthesia have less exposure to at-risk drugs, such as sevoflurane, propofol, and benzodiazepines, than patients receiving general anesthesia. At-risk drugs have been shown to negatively interfere with the neuroprotective effects of S-ketamine, which may be the reason for the failure of a large number of previous studies. There is currently a lack of randomized controlled studies evaluating S-ketamine for POD prevention, and our trial helps to fill a gap in this area.

Trial registration: http://www.chictr.org.cn, identifier ChiCTR2300075796.

## 1 Introduction

### 1.1 Background and rationale

#### 1.1.1 Delirium

**Figure 1:**
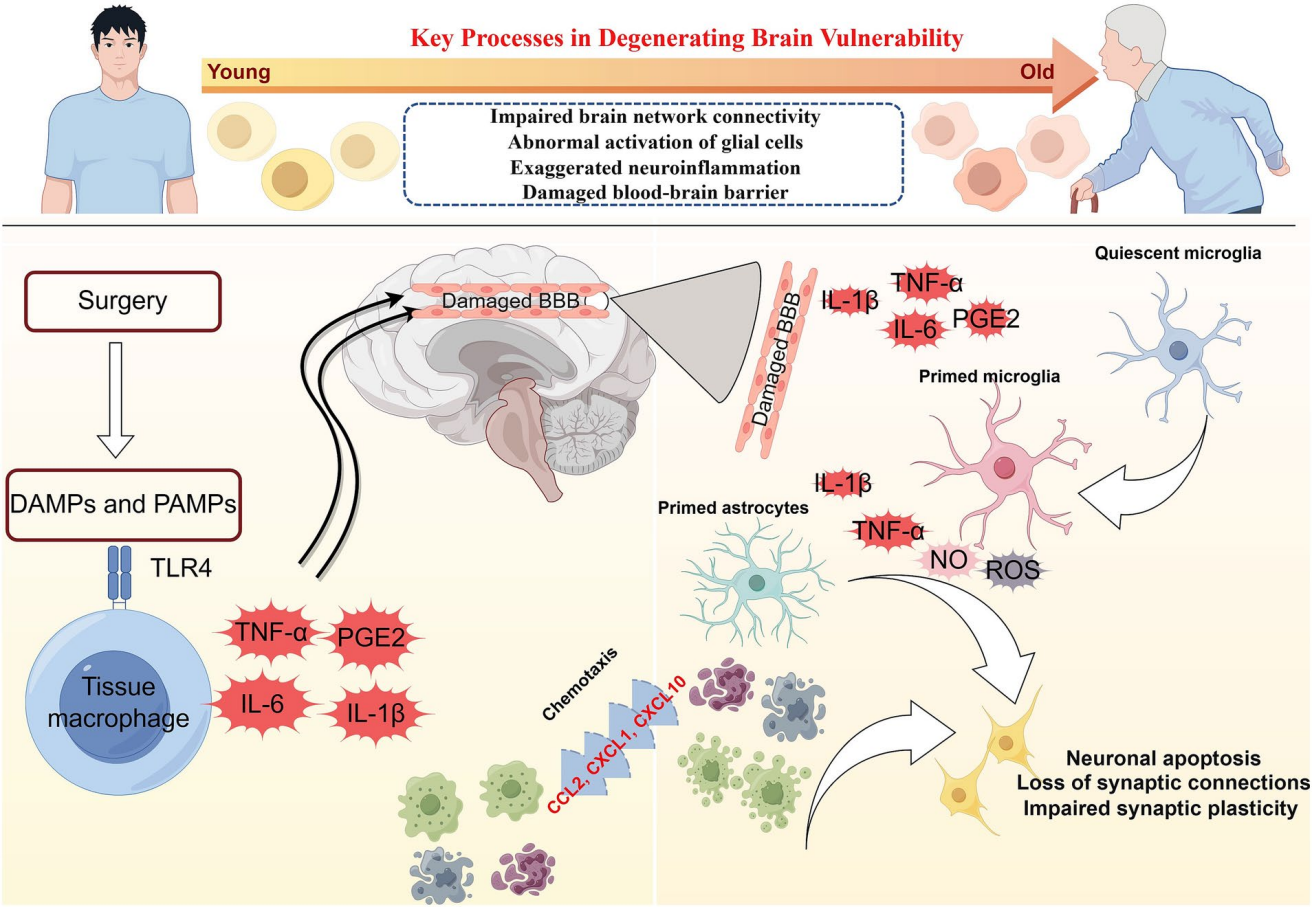
The pathophysiology of postoperative delirium caused by neuroinflammation. Surgery can induce damage-associated molecular patterns (DAMPs) and pathogen-associated molecular patterns (PAMPs). DAMPs and PAMPs mediate the secretion of interleukins (IL-1β and IL-6), tumor necrosis factor-α (TNF-α), and prostaglandin E2 (PGE2) by tissue macrophages through the binding of pattern recognition receptors (toll-like receptor 4, TLR4). These mediators enter the brain parenchyma through the damaged blood–brain barrier (BBB). Interleukin, TNF-α, and PGE2 activate microglia in a quiescent state. Primed microglia release large quantities of inflammatory mediators such as IL-1β, IL-6, reactive oxygen species (ROS), and nitric oxides (NO). IL-1β and IL-6 will activate astrocytes, which release chemokines such as CCL2, CXCL1, and CXCL10 to draw monocytes and other immune cell populations into the brain parenchyma. Neuroinflammation involving microglia and astrocytes can lead to neuronal apoptosis, impaired synaptic plasticity, and dysfunctional synaptic connections.

Postoperative delirium (POD) is an acute, transient brain dysfunction that mostly occurs within 1 week of surgery. It is characterized by inattention, changes in consciousness level, and acute changes in cognitive function. The incidence of POD varies with patient population, type of surgery, and delirium assessment tools. Elderly patients are at a high risk of POD. The incidence of POD among patients aged 60 years and older is 12.0–23.8% (Silva et al., 2021). POD is independently associated with multiple adverse outcomes, including extended hospital stays, increased medical costs, and 30-day readmission rates. POD also increases perioperative and long-term mortality risk, increases the risk of dementia, and decreases cognitive function and health-related quality of life (Witlox et al., 2010; Kong et al., 2022). The pathogenesis of POD is complex. It may be caused by one (hypoxia, hypoglycemia, or cholinergic antagonism) or multiple neurobiological mechanisms. However, the vulnerability of the degenerative brain to acute stressors or precipitating factors is a key pathophysiological basis for the development of POD (Wilson et al., 2020). The degenerative brain can manifest as impaired brain network connections (Tijms et al., 2013), abnormal activation of glial cells (Hennessy et al., 2015), exaggerated neuroinflammation (Hennessy et al., 2015), and increased permeability of the blood–brain barrier (BBB; Sweeney et al., 2018), all of which are vital processes that contribute to vulnerability (Figure 1). The vulnerable degenerative brain is unable to quickly recover from acute stressors or precipitating factors. At the molecular and cellular levels, the mechanisms by which acute stressors or precipitating factors lead to POD include insufficient brain energy metabolism (Engel and Romano, 2004), neuroinflammation (Alam et al., 2018), neurotransmitter imbalance (Itil and Fink, 1966), changes in neuroanatomic integrity (van Montfort et al., 2019), and impaired neuronal network connectivity (van Montfort et al., 2019).

#### 1.1.2 Neuroinflammation

Neuroinflammation is thought to play an essential role in POD (Figure 1; Maclullich et al., 2008). Damage-associated molecular patterns (such as high mobility group box-1, HMGB1) and pathogen-related molecular patterns (such as lipopolysaccharide) trigger the activation of tissue macrophages, which, by expressing pattern recognition receptors such as toll-like receptor 4 (TLR4), bind to the damage/pathogen-associated molecular patterns and mediate the production and release of IL-1, IL-1β, IL-6, tumor necrosis factor-α (TNF-α), and prostaglandin E2 (PGE2; Yu et al., 2006; Satoh and Akira, 2016). Inflammatory mediators can diffuse into the brain parenchyma through concentration gradients, but only to a limited extent (Banks et al., 2002). Inflammatory mediators can interact directly with the cerebral vascular system, stimulating brain endothelium and perivascular macrophages to secrete cytokines, chemokines, and prostaglandins (Cunningham, 2013). However, the entry of inflammatory mediators into the brain through the impaired BBB may be a pivotal pathway to inducing neuroinflammation. Inflammatory mediators enter the brain parenchyma through the damaged BBB to activate microglia and produce proinflammatory cytokines (IL-1β and TNF-α), reactive oxygen species (ROS), and nitric oxides (NO; Feng et al., 2017). Proinflammatory cytokines further activate astrocytes and produce chemokines (such as CCL2, CXCL1, and CXCL10) that help recruit monocytes and other immune cell populations to the brain, where they cause cognitive dysfunction or brain injury (Andonegui et al., 2018; Singh, 2022). Inflammatory mediators driven by microglia can directly affect neuronal function, leading to cell dysfunction, injury, or death. Multiple pathways (not limited to those mentioned above) can lead to neuronal damage or apoptosis, which may contribute to delirium and long-term cognitive decline.

#### 1.1.3 Rationale for ketamine in the prevention of postoperative delirium

Ketamine is an N-methyl-D-aspartate receptor (NMDA) antagonist with sedative and analgesic effects. Berman et al. (2000) made a breakthrough finding that ketamine at a subanesthetic dose (0.5 mg/kg) has a rapid antidepressant effect that lasts for at least 72 h. The rapid antidepressant effect of ketamine is achieved by inducing neuroplasticity, and synaptogenesis and synaptic potentiation are critical to this process (Zanos and Gould, 2018). Ketamine preferentially blocks calcium influx and inhibits γ-aminobutyric acid (GABA) release by acting on the NMDA receptors expressed on inhibitory GABAergic interneurons. GABA’s inhibition of pyramidal neuron activity is curtailed, significantly increasing excitatory glutamatergic neurotransmission. Glutamate activates α-amino-3-hydroxy-5-methyl-4-isoxazole-propionic acid receptors (AMPARs) on the postsynaptic membrane and promotes the release of brain-derived neurotrophic factor (BDNF). BDNF activates the downstream mammalian target of rapamycin (mTOR) signaling pathway by binding to the tropomyosin receptor kinase B (TrkB) receptor, promoting synaptic signaling protein synthesis and generation of dendritic spines, thereby enhancing connectivity in the cortex (Maeng et al., 2008; Li et al., 2010; Zanos and Gould, 2018). Ketamine can also inactivate eukaryotic elongation factor 2 kinase (eEF2K), reducing eEF2 phosphorylation, up-regulating BDNF expression levels, and promoting synaptic growth (Monteggia et al., 2013). Similar to depression, POD is associated with a loss of dendritic spines that leads to a disruption of functional brain connectivity. Neuroinflammation is thought to play an essential role in POD, and ketamine can reduce the inflammatory response by inhibiting the function of immune cells (such as lymphocytes and phagocytes; Forget et al., 2010). Low-dose ketamine also decreases the levels of the proinflammatory factors IL-6, HMGB1, NO, TNF-α, and IL-1β (Clarke et al., 2017; Luggya et al., 2017). In general, the neuroprotective effects of ketamine may be achieved by inducing the release of BDNF to promote neurogenesis and synaptic plasticity (Yan and Jiang, 2014). It can also be achieved by inhibiting the secretion of proinflammatory cytokines, thereby reducing neuroinflammation, neuronal injury, and apoptosis (Yan and Jiang, 2014).

#### 1.1.4 Clinical evidence and pharmacological deficiencies of ketamine in preventing postoperative delirium

Clinically, ketamine has been shown to prevent acute postoperative delirium in children (Chen et al., 2023). Ketamine exerts neuroprotective effects that may also potentially prevent POD in elderly patients. This hypothesis has inspired several trials to test the impact of subanesthetic doses of ketamine on the risk of POD (Urban et al., 2008; Hudetz et al., 2009; Avidan et al., 2017; Perbet et al., 2018; Plyler et al., 2019; Shurtleff et al., 2020; Hollinger et al., 2021; Zhou et al., 2021; Table 1). Hudetz et al. (2009) showed that ketamine reduced the incidence of POD by 27% in cardiac surgery patients who underwent cardiopulmonary bypass, with concomitant anti-inflammatory effects. However, two large-sample, international, multicenter randomized controlled trials showed that ketamine did not reduce the risk of POD and increased the incidence of adverse reactions such as hallucinations and nightmares (Avidan et al., 2017; Hollinger et al., 2021). A meta-analysis of randomized controlled trials that used perioperative ketamine to prevent POD and neurocognitive impairment showed that ketamine did not significantly affect the incidence of POD compared with a placebo (Hovaguimian et al., 2018; Souad et al., 2023; Viderman et al., 2023). But to be clear, ketamine did not increase the risk of POD (Reisinger et al., 2023). The negative results of these prior clinical trials are in stark contrast to the relatively solid theoretical support for the efficacy of ketamine in the prevention of POD. Ketamine’s failure to prevent POD clinically may be due to several reasons: (1) Other drugs that affect glutamate release may counteract the effects of ketamine on neuroplasticity. A classic example of this is the increased inhibitory tone of GABAergic interneurons caused by benzodiazepines (BZDs; Downing et al., 2005). The impact of BZDs on GABAergic interneurons is therefore the opposite to that of ketamine. BZDs are frequently used in clinical anesthesia as an adjunct to sedation to prevent intraoperative awareness and to counter the psychotomimetic side effects of ketamine. However, one study has shown that the intensity of psychotomimetic symptoms during ketamine infusion is positively correlated with the sustained antidepressant effects mediated by the long-term induction of neuroplasticity (Sos et al., 2013). Isoflurane, sevoflurane, and propofol also act primarily through GABAergic activation (Jackson et al., 2016). GABAergic agonists may therefore negatively interfere with the therapeutic effects of those diseases based on the neuroplastic effects of ketamine, such as depression or POD (Andrashko et al., 2020). (2) Ketamine is administered at a dose that is insufficient to prevent POD. Most studies that have evaluated the rapid antidepressant effects of ketamine used an injected dose of 0.5 mg/kg and a duration of 40 min (Xu et al., 2016). In the two large, international, multicenter controlled trials that were mentioned earlier, a single intravenous injection of ketamine was used to evaluate its ability to prevent POD. Whether a single dose of ketamine can induce similar BDNF/mTOR-mediated neuroplasticity changes to achieve the desired clinical effect of preventing POD requires further investigation. (3) The timing of ketamine may not be appropriate to prevent POD. Most of the clinical trials that used ketamine to prevent POD administered the drug during anesthesia induction or/and surgery. Only one study used ketamine postoperatively (Table 1). The effects of ketamine during anesthesia induction and/or surgery may be negatively interfered with by other GABAergic agonists, thereby diminishing or counteracting its neuroprotective effects. Ketamine may exert a better clinical neuroprotective effect when the climax of the ketamine-induced neuroplasticity cascade coincides with the neurotoxic response caused by surgery and anesthetic drugs. The administration of ketamine 1 day before or 1 day after surgery should be considered (Horacek et al., 2023).

**Table 1:** Clinical evidence for ketamine/S-ketamine for POD prevention.

Study/Type	Intervention/Control	Surgery/Patient (N)	Dose, mode, and time of ketamine/S-ketamine	Outcome/Result^§^
Hudetz (Hudetz et al., 2009)/RCT	Ketamine/Saline	Cardiac Surgery/*N* = 58	0.5 mg/kg intravenous bolus during anesthesia induction	Primary/Positive
Hollinger (Hollinger et al., 2021)/RCT	Ketamine/ haloperidol/ Ketamine+ haloperidol	Elective or emergency surgery/*N* = 182	1 mg/kg intravenous bolus before the induction of anesthesia	Primary/Negative
Avidan (Avidan et al., 2017)/RCT	Ketamine/Saline	Major cardiac and non-cardiac surgery/*N* = 672	0.5 mg/kg or 1 mg/kg intravenous bolus after the induction of anesthesia and before surgical incision	Primary/Negative
Urban (Urban et al., 2008)/RCT	Ketamine/Saline	Spinal fusion surgery/*N* = 24	0.2 mg/kg intravenous bolus after the induction of general anesthesia and 2 mcg kg^−1^ h^−1^ for the next 24 h	Secondary/Negative
Zhou (Zhou et al., 2021)/RCT	Ketamine/Saline	Brain tumor resection/*N* = 84	0.5 mg/kg intravenous bolus at the time of dural opening over 40 min	Secondary/Negative
Plyler (Plyler et al., 2019)/PPP^*^	Ketamine/Saline	Spinal fusion surgery/*N* = 30	0.5 mg/kg intravenous bolus during anesthetic induction and 0.12 mg/kg/h continuous infusion	Primary/Positive
Perbet (Perbet et al., 2018)/RCT	Ketamine/Saline	Mechanically ventilated patients/*N* = 162	0.2 mg/kg/h continuous infusion with RASS maintained at – 2-0	Secondary/Positive
Shurtleff (Shurtleff et al., 2020)/Cohort study	Ketamine/Saline	Mechanically ventilated patients/*N* = 79	0.3–1.5 mg/kg/h continuous infusion with RASS maintained at −2-0	Secondary/Negative
Ma (Ma et al., 2023)/RCT	S-ketamine/Saline	Gastrointestinal Tumors/*N* = 62	0.25 mg/kg intravenous bolus during anesthetic induction and a 0.125 mg/kg/h continuous infusion until 20 min before the end of surgery	Secondary/Negative
Bornemann (Bornemann-Cimenti et al., 2016)/RCT	S-ketamine/Saline	major abdominal surgery/*N* = 60	Low-dose group: 0.25 mg/kg intravenous bolus at the time of general anesthesia induction followed by 0.125 mg kg^−1^ h^−1^ for 48 h Minimal-dose group: 0.015 mg kg^−1^ h^−1^ continuous infusion for 48 h	NA

POD, postoperative delirium; RCT, randomized controlled study; PPP^*^, prospective preimplementation and postimplementation design; RASS, Richmond Agitation-Sedation Scale; Outcome/Result^§^, Outcome is defined as POD is the primary or secondary outcome. Result is defined as the difference in POD incidence between two or more groups. Positive means statistically significant difference, negative means none; NA: It is unclear whether POD is a primary or secondary outcome, and the difference in POD incidence between the two groups is unclear.

#### 1.1.5 Current status of S-ketamine in the treatment of postoperative delirium

S-ketamine is a dextrorotatory substance that is isolated and purified from ketamine. Its affinity to the NMDA receptor is 3–4 times that of R-ketamine, and its analgesic effect is 2–2.5 times that of ketamine. S-ketamine has a high clearance rate *in vivo* and causes fewer psychotropic side effects. Some indirect evidence, such as animal studies (Himmelseher et al., 2000; Treccani et al., 2019), healthy volunteer trials (Höflich et al., 2021), and human metabolomic studies (Nummela et al., 2022), suggests that S-ketamine may help to improve depression and cognitive impairment by exerting a neuroprotective effect. However, few clinical studies have evaluated the utility of subanesthetic doses of S-ketamine to prevent POD or cognitive dysfunction. Han et al. (2023) showed that 0.15 mg/kg IV S-ketamine administered 5 min before the start of surgery significantly reduced the incidence of delayed neurocognitive recovery (DNR) in patients undergoing gastrointestinal surgery. Unfortunately, this study did not focus on the effect of S-ketamine on POD. Ma et al. (2023) showed that 0.25 mg/kg IV S-ketamine administered during anesthesia induction followed by continuous IV administration at 0.125 mg/kg/h (infusion stopped 20 min before the end of surgery) significantly reduced the incidence of DNR in patients with gastrointestinal tumors, but had no significant impact on the incidence of POD. Bornemann-Cimenti et al. (2016) showed that a minimal dose of S-ketamine (0.015 mg/kg/h continuous infusion for 48 h) in patients who underwent major abdominal surgery resulted in a lower Intensive Care Delirium Screening Checklist score than a low dose of S-ketamine (0.25 mg/kg loading dose, 0.125 mg/kg/h continuous infusion for 48 h) or a placebo. The assessment tools, outcome measures, administration method of S-ketamine, and small sample size of the two aforementioned clinical studies were insufficient to confirm the effects of S-ketamine on POD.

### 1.2 Objectives

To date, most trials that have evaluated the utility of subanesthetic doses of ketamine in the prevention of POD have not yielded supportive results. Given the severe harm that POD brings to the health of elderly patients and the potential that S-ketamine’s ability to prevent POD is inhibited by how we are presently using it, we believe that further studies are necessary to understand the potential of S-ketamine in this indication. We therefore designed this prospective, double-blind, placebo-controlled trial to explore the potential role of S-ketamine in the prevention of POD.

## 2 Methods

### 2.1 Design and setting

**Figure 2:**
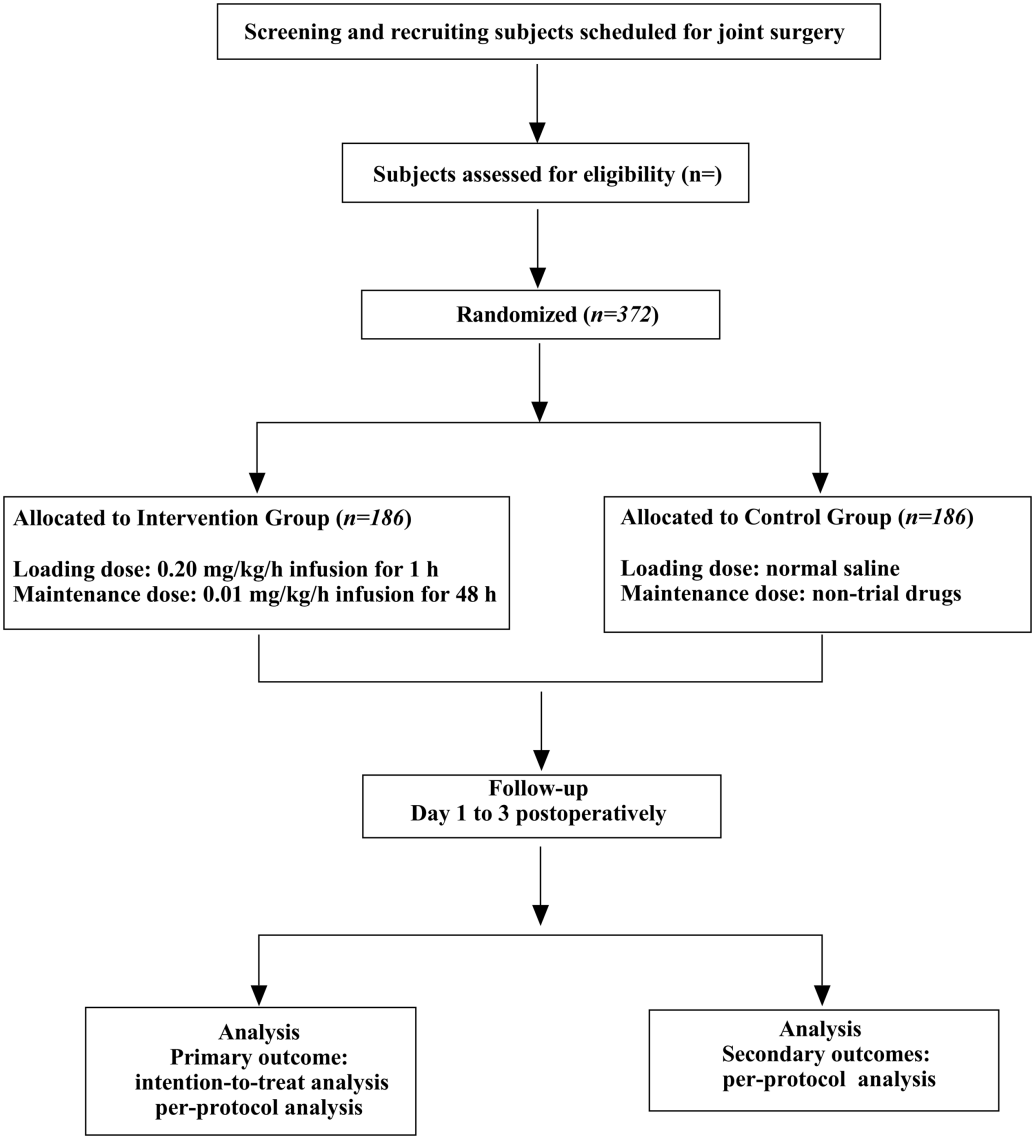
Consolidated standards of reporting trials (CONSORT) flow diagram.

This is a single-center, randomized, double-blind, placebo-controlled, pragmatic study. The trial will be conducted at The Affiliated Hospital of Qingdao University. Eligible patients will be randomly assigned to intervention and control arms at a 1:1 ratio (Figure 2).

### 2.2 Eligibility criteria

#### 2.2.1 Inclusion criteria

The inclusion criteria will be patient age ≥ 60 years old; selective total hip or knee arthroplasty (THA or TKA) under intraspinal anesthesia; and signed informed consent.

#### 2.2.2 Exclusion criteria

This trial is a pragmatic study, so exclusion criteria will not be strictly defined. This means that patients with preoperative dementia and depression will not be excluded. However, we plan to exclude: (1) patients who are delirious and psychotic before the start of the trial; (2) patients with contraindications to S-ketamine (such as allergies, intracranial aneurysms, hyperthyroidism, and glaucoma); (3) patients with severe cardiovascular disease (e.g., hypertension grade III, unstable angina pectoris, severe valvular heart disease, severe arrhythmia, severe macrovascular disease); (4) patients with severe hepatic or renal insufficiency (such as Child-Pugh score III, creatinine clearance rate < 35 mL/min); (5) patients with a substance abuse history (such as ketamine, cocaine, heroin, amphetamine, and methamphetamine); (6) patients taking psychotropic drugs (such as chlorpromazine, clozapine, olanzapine, risperidone, and quetiapine); (7) family history of malignant hyperthermia or patients at high risk for malignant hyperthermia (such as strabismus and scoliosis); (8) body mass index (BMI) ≤ 18 kg/m^2^ or > 30 kg/m^2^.

#### 2.2.3 Drop-out criteria

A subject may be withdrawn from the trial for the following reasons: (1) the investigator considers it necessary to stop the trial due to a medical ethics issue; (2) the incidence of a severe adverse event; (3) withdrawal from the study is felt by the investigator to be in the best interest of the subject; (4) poor compliance; (5) the subject has the right to withdraw from the trial midway through, or may not withdraw their informed consent but choose not to receive a study intervention. The reason for the subject’s withdrawal from the trial will be recorded on a “Withdrawal/Change of Status” form.

### 2.3 Sample size calculation

The sample size for this trial is based on the incidence of POD. A previous meta-analysis reported a 17% incidence of POD in patients who underwent a total joint arthroplasty (Scott et al., 2015). When considering the potential effect of the study intervention, we used the absolute risk reduction of 10% (corresponding to a number needed to treat of 10 patients) recommended by previous studies (Avidan et al., 2017). We therefore assumed that the incidence of POD in the intervention group would be 7%. We used Power Analysis and Sample Size version 15.0 (Stata Corp. LP, College Station, TX, United States) to calculate a sample size of 166 per group. Considering a 10% lost to follow-up rate, at least 186 subjects in each group and 372 subjects should be included.

### 2.4 Recruitment

This is a single-center clinical trial, and we plan to recruit subjects at The Affiliated Hospital of Qingdao University. The recruitment method is a combination of on-site recruitment and open recruitment. The surgeon may invite eligible subjects to participate in this trial. The subject recruitment team will also use posters and leaflets to invite patients to participate in the clinical trial.

### 2.5 Randomization and allocation concealment

Subjects will be assigned a trial number (1–372) in the order in which they are registered. We will use a block randomization method to randomly assign subjects to the intervention and control groups in a 1:1 ratio with variable block lengths (2, 4, 6, 8). The randomization scheme will be the responsibility of a dedicated person (Xin Jiang) from the clinical trial center who will not be involved in other parts of the trial. The randomization scheme will be placed in a sealed, opaque envelope. The quality controller (Fang Sheng) will open the envelopes in the order of their enrollment before the start of the trial, and subjects will be grouped according to the allocation scheme in the envelope. The quality controller will not participate in other parts of the trial.

### 2.6 Double blinding

This study is double-blinded. According to the allocation scheme, subjects will receive an intravenous infusion of S-ketamine or 0.9% saline during the procedure and a continuous infusion of S-ketamine or 0.9% saline via patient-controlled intravenous analgesia (PCIA) device after surgery. To ensure that the investigators are blinded, the drug administrator (Yan Han, Mingxia Shan) will prepare two 20 mL syringes (Weigao Group Medical Polymer Products Co., Ltd.) in a separate room filled with S-ketamine at a concentration of 2.0 mg/mL or 0.9% normal saline. The drug administrator will also configure the PCIA device according to the allocation scheme of the subject. The syringes and the PCIA device will look the same. The drug administrator will not be involved in other parts of the trial. Nurses, surgeons, anesthesiologists, outcome assessors, and statistical analysts will be blinded to the subject’s group and intervention throughout the trial. An independent data and safety monitoring board will oversee the practice and will not disclose the allocation group of the subjects until statistical analysis is completed. In an emergency (such as rapid deterioration of the patient’s clinical status), the anesthesiologist may request disclosure of the intervention the subject received or adjust or interrupt the study drug infusion if necessary, which will be documented. Unblinded subjects will be included in the intention-to-treat (ITT) analysis but excluded from the per-protocol (PP) analysis.

### 2.7 Baseline assessment

This is a pragmatic clinical trial, so we will identify confounding factors that may impact the primary outcome. The study team (Qun Li, Xue Sun) will visit the patient before surgery and perform a health assessment. The study team will document the subject’s baseline characteristics by consulting the Hospital Information System and via face-to-face interviews. Baseline characteristics that will be collected include the subject’s age, sex, American Society of Anesthesiologists (ASA) physical status classification system (I [healthy], II [mild systemic disease], III [serious systemic disease], and IV [serious systemic disease with persistent life-threatening condition]); BMI, education level (<elementary school, elementary school, and ≥ secondary school), comorbidities (cardiovascular system, central nervous system, respiratory system, urinary system, gastrointestinal system, and hematologic system), electrolyte level (any concentration of potassium, sodium, calcium and magnesium ions above or below the normal range is defined as abnormal), albumin and hemoglobin concentration, smoking status (smoking is defined as continuous or cumulative smoking for more than 6 months or smoking at least 1 cigarette per day and currently smoking. Subjects will otherwise be defined as never smoking), alcohol consumption (alcohol consumption is defined as an average of more than 60 grams of pure alcohol per day for men and more than 40 grams of pure alcohol per day for women), history of falls (falls within the past 6 months), the presence of vision or hearing abnormalities, preoperative pain scores at rest and exercise, and baseline measurements of mental state, attention, thinking, and consciousness levels using the Chinese version of 3D-CAM.

The study team (Lili Jiang, Zhijin Zou) will use a Mini-Mental State Examination (MMSE) scale to assess the subjects’ preoperative cognitive function. Dementia will be defined according to the patient’s education level and MMSE (illiteracy <18 points, primary education <20 points, secondary education <23 points, and postsecondary education <24 points). The Patient Health Questionnaire-9 (PHQ-9) evaluates the subject’s mood. A score of 0–4 is normal, while ≥5 is considered diagnostic for depression. The depression screening needs to be combined with the subject’s medical and medication history. The subject’s functional status will be evaluated with the Barthel index (BI), with a score ≥ 40 considered good functional status and a score < 40 considered poor functional status. The frailty status of the subject will be assessed using The FRAIL Scale, with a score of ≥3 considered frailty and a score of <3 considered no frailty. The Pittsburgh Sleep Quality Index will be used to evaluate the sleep quality of the subject over the past 1 month, with a score of 0–7 considered for no sleep problems and a score of 8 for a sleep disorder. The age-adjusted Charlson Comorbidity Index (aCCI) will be used to assess the comorbidities of the subjects.

### 2.8 Anesthesia and perioperative analgesia

Subjects received oral celecoxib 200 mg (Pfizer pharmaceutical co., Ltd., Dalian, Liaoning, China) once daily prior to surgery. Subjects will be transferred to the anesthesia induction room on the day of surgery. Peripheral venous access will be obtained and 3-lead ECG, noninvasive blood pressure, and oxygen saturation monitoring will be established. Depending on the type of procedure, each subject will undergo either an ultrasound-guided adductor canal block or an iliac fascial block before anesthesia induction. Twenty minutes after the block, the subject will be transported to the operating room for intraspinal anesthesia. As this is a pragmatic clinical trial, the use of anesthetic drugs and techniques (epidural vs. subarachnoid block) is at the discretion of the anesthesia team that is assigned to each subject. The anesthesiologist can choose the appropriate medication regimen according to their clinical practice. However, the anesthesia team needs to use the study drug according to the guidance of the drug administrator. The team will also be asked to avoid the use of benzodiazepines, dexmedetomidine, S-ketamine, and other NMDA receptor antagonists. During the surgery, the patient’s body temperature will be continuously monitored with a body temperature monitor to maintain their body temperature at 36°C–37°C. Subjects with hypothermia will be treated with a warm air blower. Infusion and transfusion will be performed according to routine procedures, and blood pressure will be maintained within 20% of baseline. Twenty minutes before the end of surgery, the anesthesiologist will administer ondansetron 4 mg (Qilu Pharmaceutical Co., Ltd., Jinan, Shandong, China) to prevent nausea and vomiting. The PCIA device will be activated in the post-anesthesia care unit (PACU). When the subject’s Number Rating Score (NRS) rises above 4 points, they will receive intravenous fentanyl 50 μg (Jiangsu Enhua Pharmaceutical Co., Ltd., Xuzhou, Jiangsu, China) as rescue analgesia. Subjects will be transferred to the surgical ward when they meet the transfer criteria. Subjects will receive flurbiprofen axetil 50 mg (Beijing Taide Pharmaceutical Co., Ltd., Beijing, China) twice daily as basic analgesic therapy. When the subject’s NRS rises ≥4, they will receive an intravenous injection of 50 mg pethidine (Qinghai Pharmaceutical Factory Co., Ltd., Xining, Qinghai, China), which can be repeated every 4–6 h, with a maximum dose of 300 mg per day. Rescue analgesia is adequate when the subject’s NRS is <4 points. In the case of moderate to severe postoperative nausea or vomiting, ondansetron 0.1 mg/kg will be administered intravenously.

### 2.9 Interventions

Subjects in the intervention group will receive an intravenous infusion of S-ketamine (Jiangsu Heng Rui Medicine Co., Ltd., Jiangsu, China) following a standard dilution protocol (40 mg S-ketamine with 0.9% normal saline to 20 mL, 2.0 mg/mL). The infusion rate of S-ketamine will be 0.1 mL/kg/h (0.20 mg/kg/h), the infusion mode will be constant speed pumping, and the infusion time will be 1 h. In the control group, subjects will receive 0.9% saline at 0.1 mL/kg/h for 1 h at a constant rate infusion.

All subjects will be transferred to the PACU after surgery. The PCIA device will be connected and started. In the intervention group, the PCIA device will be filled with 1.0 mg/kg S-ketamine, 50 μg sufentanil (Yichang Renfu Pharmaceutical Co., Ltd., Yichang, Hubei, China), 6 mg butorphanol tartrate (Jiangsu Heng Rui Medicine Co., Ltd., Jiangsu, China) and 8 mg ondansetron, diluted to 100 mL with normal saline. In the control group, the PCIA device will not contain S-ketamine. The background dose will be 1 mL/h, the single bolus dose 2 mL, the lockout time 15 min, and the maximum infusion rate 8 mL/h.

The choice of S-ketamine dose and mode of administration was based on published clinical trials and drug manufacturer instructions. S-ketamine is twice as potent in analgesia as ketamine, and the same anesthetic effect can be achieved with a dose of 1/2 ketamine. A subanesthetic dose of S-ketamine (0.25 mg/kg) was often selected by the investigator for the prevention of POD or perioperative neurocognitive dysfunction (Wei et al., 2022; Han et al., 2023; Ma et al., 2023). However, we need to be clear that a dose of 1/2 ketamine may not be equivalent to the dose at which S-ketamine exerts neuroprotective effects, and that relatively high doses of S-ketamine are more prone to adverse events and potential safety concerns (McIntyre et al., 2021). Subjects will receive intraspinal anesthesia rather than general anesthesia in this study, which avoids the influence of other anesthetic drugs on the neuroprotective effects of S-ketamine. In addition, elderly patients with preoperative dementia, depression, and multiple comorbidities will be included, making the dose of S-ketamine received in the intervention group more conservative. Our previous study found that subjects receiving 0.3 mg/kg/h S-ketamine had hallucinations and dreaminess, although this dose was subanesthetic (Zhu et al., 2023). Therefore, we will use a smaller subanesthetic dose of S-ketamine 0.2 mg/kg infused over 1 h in this trial. In antidepressant therapy, the short-term efficacy of intravenous ketamine necessitates repeated dosing in most cases to maintain therapeutic benefit. Therefore, the intervention group will receive an intravenous infusion of 0.010 mg/kg/h for 48 h via the PCIA device after surgery, a dose with fewer psychiatric side effects and maintaining the need for analgesia.

### 2.10 Standardized training for delirium assessors

All study team members performing delirium assessments will undergo a rigorous, standardized training process. Three psychiatrists will train the delirium assessors before the start of the study. The training is divided into three parts: the exercise of theoretical knowledge of delirium, simulation training to perform delirium assessments, and an examination. Training in the theoretical understanding of delirium includes the definition, clinical manifestations, and classification of delirium, delirium evaluation tools (3D-CAM and CAM-S), and the differential diagnosis of delirium. The simulation training program for delirium assessment is a 7-day training program led by three psychiatrists. Evaluators will first view three standard interview videos and then use 3D-CAM and CAM-S to score the content described in the video to assess the presence and degree of delirium. Scores will then be compared with the video’s “gold standard” score to evaluate the assessor’s mastery of the delirium assessment. During the examination phase, the delirium assessment team will be divided into three groups, each consisting of two assessors and a psychiatrist. The delirium assessor must perform a simulated assessment and scoring of delirious and non-delirious patients using 3D-CAM and CAM-S. The assessor must agree with the psychiatrist on the presence or absence of all delirium features for at least three patients. During the examination phase, the assessor must demonstrate competence in delirium assessment and scoring, and the diagnosis of delirium must be in 100% agreement with the psychiatrist to be considered a pass. The training process will be repeated every month during the study to establish the assessor’s ability to score delirium reliably.

### 2.11 Outcomes

#### 2.11.1 Primary outcome

The primary outcome is the incidence of POD within 3 days of surgery. The initial evaluation of delirium will begin at least 6 h after surgery followed by 2 assessments daily (800–1,000 and 1,600–1,800) for the first 3 days after surgery, with an interval of at least 6 h between evaluations. Delirium will be assessed using the Chinese version of 3D-CAM (Marcantonio et al., 2014) in addition to medical records over the previous 24 h, nursing records, and discussions with family members.

The 3D-CAM identifies delirium through a diagnostic algorithm based on the four essential features of delirium: (A) acute changes or fluctuations in mental status; (B) inattention; (C) disorganized thinking; and (D) altered level of consciousness. Diagnosis of delirium requires criteria A and B and either or both of criteria C and D. The 3D-CAM has a high sensitivity (84–99%) and specificity (90–97%). If the subject is discharged or dies within 3 days, the last delirium assessment will be considered the replacement for any missing data.

#### 2.11.2 Secondary outcomes

1. Number of POD episodes. One positive 3D-CAM assessment can be diagnosed as POD. Two consecutive negative 3D-CAM assessments were considered a transition from POD to non-POD status.
2. The onset time and duration of delirium. The onset time of delirium is defined as the time from the subject being transferred back to the surgical ward to the first diagnosis of delirium. The duration of delirium is defined as the time elapsed from the subject’s diagnosis of delirium to 2 negative 3D-CAM assessments. The maximum duration of delirium will be recorded in patients with multiple delirium episodes.
3. Delirium severity score. Patients with established delirium will also be assessed for delirium severity using the CAM-S (Inouye et al., 2014), with daily maximum scores recorded. The CAM-S scale has 4 items with a total score of 7 points. Symptom severity can be classified as absent (0), mild (1), or marked (2). The total score of 0 is normal, 1 is mild delirium, 3–5 is moderate delirium, and 6–7 is severe delirium.
4. Subtypes of delirium. Delirium can be divided into three motoric subtypes according to its clinical manifestations: hyperactive, hypoactive, and mixed. The Richmond Agitation-Sedation Scales (RASS) classify delirium subtypes in patients with established delirium (Ely et al., 2003). Hyperactive delirium is defined as RASS that are consistently positive (+1 to +4); hypoactive delirium is defined as RASS that are consistently neutral or negative (−3 to 0); mixed delirium is defined when some RASS are positive (+1 to +4) and some are neutral or negative (−3 to 0).
5. Pain scores at rest and exercise. Assessments will be performed using the NRS at 8:00–10:00 on postoperative days 1–3, during physical therapy on postoperative days 1–3, and at 16:00–18:00 on postoperative days 1–3. Resting is defined as the subject being in a quiet supine position, and exercise is defined as the subject lifting the affected limb at least 5 times to a height of at least 30 cm.
6. Opioid consumption. Opioid consumption will be assessed on postoperative day 1, postoperative day 2, and overall. Opioid consumption, including PCIA and rescue analgesics, will be converted to a “morphine equivalent dose” for analysis.
7. The time of first analgesia pump compression within 24 h of surgery (defined as the time elapsed from the start of the analgesia pump to its first compression).
8. Post-operative sleep quality: The NRS will be used to assess the subject’s sleep quality on postoperative days 1–3 (on an 11-point scale where 0 = best sleep and 10 = worst sleep).
9. Preoperative baseline and 3 days postoperative Timed Up and Go test. The Timed Up and Go test is the time it takes to get up from a standard-height armchair, walk three meters forward, walk back to the chair, and sit down.
10. The active range of motion of the affected knee or hip at baseline and 3 days after surgery. The active range of motion is defined as the range from neutral (0°) to maximum flexion.
11. BI score at baseline and 3 days after surgery.
12. Time to discharge criteria, which include the subject being able to walk independently from their bed to the bathroom and along a hallway without the aid of a walker.
13. Length of hospital stay. It is defined as the time elapsed from the day of the subject’s procedure to discharge.

#### 2.11.3 Safety outcomes

Patients will be monitored for adverse events for 24 h after surgery or until the resolution of an adverse event. Tachycardia is a heart rate greater than 100 beats per minute and bradycardia is less than 45 beats per minute. Hypertension is a systolic blood pressure greater than 180 mmHg or a greater than 20% increase from baseline, and hypotension is systolic blood pressure less than 90 mmHg or a greater than 20% decrease from baseline. Hypoxemia is a blood oxygen saturation of less than 95% with supplemental oxygen and a pulse oxygen saturation of less than 90% without supplemental oxygen. Unplanned transfer to ICU. S-ketamine-related psychiatric complications include dreaminess, nightmares, dizziness, and hallucinations. Early postoperative nausea and vomiting refers to nausea and vomiting within 2 h of surgery. A non-delirious postoperative complication is a new illness other than postoperative delirium requiring therapeutic intervention within 30 days of surgery.

### 2.12 Trial safety

The study team will give the subjects or their designated agents a complete and comprehensive introduction to the study objectives, study procedures, investigational drugs, reasonably expected benefits, possible toxicities, and the risks of participating. Written informed consent will be obtained from all subjects to protect their legitimate rights and interests. The study team must improve an emergency plan before the start of the trial. This will involve communication with multiple disciplines to inform them of the possible adverse events that may occur during this clinical study and to obtain their assistance in case of an emergency. The dose and concentration of S-ketamine that will be received by subjects in this trial were selected based on the pharmacokinetics, pharmacodynamics, published clinical trials, and recommendations from the drug’s manufacturer. Interventions for tachycardia, bradycardia, hypotension, and hypertension include adjustment of the study drug infusion, administration of vasoactive agents, or both. Interventions for hypoxemia include oxygen administration or physical therapy. Management of subjects with POD should first include identifying and correcting the underlying etiology, including the immediate exclusion of life-threatening causes such as hypoxia, hypotension, hypoglycemia, and sepsis. Non-pharmacologic treatment is the first-line treatment strategy. Haloperidol will be administered to subjects with severe agitation (RASS score of +3 or greater) who fail to respond to non-medical therapy. The study team will treat or rescue drug-induced adverse reactions such as nausea, vomiting, and anaphylactic shock according to established standard operating procedures. The investigators involved in this trial have extensive clinical experience, are good at handling acute and critical events, and have received professional Good Clinical Practices (GCP) training. The multifunction monitor will monitor the subject during the procedure and in the PACU. Subjects will be monitored continuously in the surgical ward for at least 24 h using ECG, blood oxygen levels, and blood pressure.

### 2.13 Adverse events reporting

Adverse events (AE) refer to all untoward medical events that occur after the subject receives the investigational product. These include symptoms, signs, diseases, or laboratory abnormalities but may not necessarily have a causal relationship with the investigational product. The investigator should record and report all AE that are directly observed or spontaneously written by the subject in concise language. In addition, subjects should be asked about AE from the beginning to the end of the trial. AE should be recorded on the designated Adverse Event Form. The severity of AE will be judged according to standard adverse reaction evaluation criteria. All AE will be handled according to the standard operating procedures for handling adverse reactions that were developed by the clinical trial center. The AE record form shall be truthfully filled in, including the occurrence time, severity, duration, measures taken, and outcome of the AE.

A serious adverse event (SAE) refers to death or a life-threatening event, permanent or severe disability or loss of function, hospitalization or prolongation of hospitalization, a congenital anomaly or disability, or any other important medical events experienced by the subject after receiving the investigational product. Any subject with SAE should be withdrawn from the trial, and the investigator should actively treat the subject with SAE. The investigator must also complete the SAE Report Form in as much detail as possible, and sign and date it. The investigator should report the SAE to the Principal Investigator (Yang Zhao) and the Ethics Committee of Qingdao University within 24 h of being informed of the SAE.

### 2.14 Data collection

Data will be collected via face-to-face interviews with subjects and relatives or extracted from the HIS (Table 2).

**Table 2:** Enrollment schedule, interventions, and assessments.

Outcome measure	Visit 1	Visit 2	Visit3	Visit4	Visit 5	Visit 6	Visit 7	Visit 8
Time	Day of admission	1 day pre-surgery	During operation	6 h post-operative	1 d post-operative	2 d post-operative	3 d post-operative	Hospital discharge
Eligibility screening	X		–	–	–	–	–	–
Informed consent	X		–	–	–	–	–	–
Demographic characteristics	–	X	–	–	–	–	–	–
Baseline measures	–	X	X	–	–	–	–	–
Randomization	X	–	–	–	–	–	–	–
Intervention	Intraoperative S-ketamine will be infused continuously at 0.20 mg/kg/h for 1 h, followed by 0.01 mg/kg/h for 48 h							
Incidence of POD	–	–	–	X	X	X	X	–
Onset time of POD	–	–	–	X	X	X	X	–
Duration of POD	–	–	–	X	X	X	X	X
Severity of POD	–	–	–	X	X	X	X	–
Subtypes of POD	–	–	–	X	X	X	X	–
NRS	–	–	–	–	X	X	X	–
Opioid consumption	–	–	–	–	X	X	–	–
Pump compression	Within 24 h of surgery							
Clinical outcomes	–	X	–	–	–	–	X	–
Length of stay	–	–	–	–	–	–	–	X
Safety outcomes	Throughout the study period until discharge							

POD, postoperative delirium; NRS, number rating score.

#### 2.14.1 Preoperative data

1. Age (years), gender, ASA classification (I-IV), education level (<elementary school, elementary school, ≥secondary school), BMI (kg/m^2^), and comorbidities.
2. Electrolyte abnormalities (yes/no), low protein (yes/no), and anemia (yes/no).
3. Smoking (yes/no), alcohol use (yes/no), history of falls (yes/no), abnormal vision or hearing (yes/no), dementia (yes/no), depression (yes/no), functional status (self-care/need help or dependence), frailty status (yes/no), sleep disorder (yes/no), and aCCI.
4. Preoperative baseline Timed Up and Go test (s), preoperative baseline active range of motion (°) of the affected knee or hip, and preoperative baseline BI score.

#### 2.14.2 Intraoperative data

1. Type of surgery (TKA/THA), surgery time (min), anesthesia time (defined as time elapsed from anesthesia induction to the end of surgery, min).
2. Blood loss (mL), infusion volume (defined as the total volume of crystalloid and colloid, mL), and transfusion volume (mL).
3. Steroid dosing (mg).

#### 2.14.3 Postoperative data

1. Incidence of POD from 6 h to 3 days after surgery, the onset and duration of the POD, the POD severity score, and the subtype of POD.
2. Pain scores during rest and exercise.
3. Opioid consumption (written as morphine equivalent dose, mg).
4. Sleep quality on postoperative days 1–3.
5. Clinical outcomes: Timed Up and Go test (s), active range of motion of the affected knee or hip (°), BI score, time to discharge criteria (d), and length of hospital stay (d).

#### 2.14.4 Safety data

1. The incidence of tachycardia, bradycardia, hypertension, hypotension, hypoxemia, and unplanned ICU transfer.
2. S-ketamine-related psychiatric complications such as dreaminess, nightmares, dizziness, and hallucinations.
3. Early postoperative nausea and vomiting
4. Non-delirium postoperative complications.

### 2.15 Data management

This trial will use an electronic data capture system (EDC) for data management. According to the clinical study protocol, the data manager will design the electronic case report form (eCRF) and write the data management and verification plans to standardize the data management process. All trial data will be recorded in eCRF by GCP-trained study personnel designated by the investigator. After the source data information is collected, the investigator or authorized clinical coordinator will log in to the EDC through a separate account and enter the subject data according to the guidance/requirements of the completion guide. All data should be entered wholly, truthfully, accurately, and on time. The database will be backed up routinely throughout the study. The database is deployed on an Alibaba Cloud Virtual Machine and automatically backed up at a fixed time daily. According to the established verification plan, the Clinical Research Associate shall perform data verification on time. Questions identified during data verification should be answered and resolved by the study director. After confirming the data rationality, consistency, and completeness, the database will be locked and exported to the statistical analysis system. The source data and backup copies of the database will be kept in a secure and confidential location and guaranteed to be available for inspection by the Data Monitoring Committee at all times. The eCRFs and EDC database will be retained for at least 10 years.

### 2.16 Statistical methods

To analyze the primary study outcome, we will use the ITT analysis set. The ITT analysis set is defined as all subjects who undergo randomization regardless of their intervention. Data processing and analysis will be performed using R version 4.3.0 and the Storm Statistical Platform.^1^ Continuous variables will be tested for normality using Shapiro–Wilk, with normal data expressed as mean ± SD, non-normal data as median and interquartile range, and categorical data as number of cases (n) and percentage (%). For demographic data, baseline characteristics, and intraoperative data, quantitative data between the two groups will be compared using a two-independent sample t-test or Mann–Whitney U test, and qualitative data will be compared using the chi-square test, continuous corrected chi-square test, or Fisher’s exact test. The primary outcome is a dichotomous measure, and the odds ratio (OR) and 95% confidence interval (CI) of the incidence of POD between the intervention and control groups will be calculated using a logistic regression model. This is a pragmatic study, and potential confounding variables may bias the degree of association between interventions and primary outcomes. The logistic regression model will therefore include demographics and baseline characteristics as covariates. We plan to build four different models. Model 1 will only include grouping factors and not control for confounding factors. The net OR and 95% CIs will be calculated. Model 2 will adjust for sex, age, ASA classification, comorbidities, and education level. Model 3 will adjust for electrolyte abnormalities, hypoproteinemia, and anemia based on Model 2. Model 4 will adjust for smoking, alcohol use, history of falls, vision or hearing abnormalities, preoperative dementia, depression, functional status, frailty, sleep disorders, and aCCI based on Model 3. Adjusted OR and 95% CIs will be calculated from Models 2, 3, and 4. We will perform a sensitivity analysis on the primary outcome to compare the effects of different analysis sets (ITT and PP) and statistical methods (modified Poisson regression and logistic regression) on the robustness of the primary outcome. The PP analysis set excludes subjects who did not meet our eligibility criteria or had incomplete adherence to the trial medication. Subgroup analyses will be performed for age, gender, dementia, depression, frailty, sleep disorders, vision or hearing abnormalities, and functional status, and interactions will be investigated.

We will only use the PP analysis set to evaluate our secondary outcomes. Our secondary outcomes are exploratory, and no adjustment for type I errors will be made for multiple comparisons. The ordered multiclass (number of POD episodes) and disordered multiclass (subtype of POD) variables will be analyzed using the Mann–Whitney U test, chi-square test, or Fisher’s exact test. Quantitative data (onset and duration of POD, delirium severity score, time to discharge criteria, and length of stay) will be analyzed using the Mann–Whitney U test, and pseudo-median difference and 95% CIs will be estimated using the Hodges-Lehmann method. Time to first pump compression is survival data and will be analyzed using the Kaplan–Meier survival curve and log-rank tests. Hazard ratios (HR) and 95% CIs will be calculated using a one-way COX regression. NRS scores, opioid consumption, and sleep quality at different time points after surgery are repeated measures data and will be analyzed using generalized estimating equations. The difference and 95% CIs will be calculated. Analysis of covariance (ANCOVA) will be used to compare between-group differences in the Timed Up and Go test, active range of motion of the affected knee or hip, and BI score, adjusting for covariates (baseline measurements), and estimating least square mean (LSM) differences and 95% CIs. Safety indicators will be analyzed using the Safety Analysis Set, which will include all randomized subjects who received at least one dose of medication. All statistical tests will be two-sided. *p* < 0.05 will be considered statistically significant.

### 2.17 Missing data

Missing data in the ITT analysis set will be handled using the multiple imputation method, with the number of imputations set to 5 and the maximum number of iterations set to 50. Continuous variables will be imputed using a predictive mean-matching model, and categorical variables will be imputed using a logistic regression model.

### 2.18 Data monitoring

The Data Monitoring Committee (DMC) will be comprised of a clinical center director, a biostatistician, and a medical ethics committee member. The three members are independent of each other and have no conflict of interest with the trial. The DMC will verify the study protocol, informed consent form, statistical analysis plan, study conduct, and data accuracy, completeness, and timeliness according to the verification plan. Problems found during data verification should be reported online and answered by the Principal Investigator (Yang Zhao). Each query will be closed after the query publisher confirms that no error exists. If there is a problem, the query can be reissued until the issue is entirely resolved. The DMC members can recommend that the Principal Investigator continue, modify, suspend, or terminate the study.

### 2.19 Ethics and confidentiality

This trial is approved by the Medical Ethics Committee of The Affiliated Hospital of Qingdao University and registered in the China Clinical Trial Registry (identifier ChiCTR2300075796). The ethics committee must re-approve any amendment to the trial protocol that is made during the study period. Before the start of the trial, the study team will obtain written informed consent from all subjects, their family members, or their legal representatives. The investigator shall give the subject or designated agent a complete and comprehensive introduction to the purpose of this study, the study model, the drug effect, reasonable expected benefits, possible toxic and side effects, and possible risks. This clinical trial will be conducted under the Declaration of Helsinki and per Good Clinical Practice, issued by the National Medical Products Administration and relevant regulations. Each subject will be given a unique ID, and their information will be adequately protected. Subject personal details will not be disclosed unless authorization is approved.

### 2.20 Dissemination

Results will be made public after the trial is completed. The investigator will communicate the trial results to the subjects, other healthcare professionals, the general public, and any other interested group through publications or presentations at academic conferences. The study director will be considered the corresponding author, the study protocol designer will be the first author, and any investigator who has contributed to the trial for at least 4 months will be considered a co-author. Other investigators will be recognized in publications based on their contributions to the trial.

## 3 Discussion

This single-center, prospective, double-blind, randomized, placebo-controlled, pragmatic study will assess our hypothesis that elderly patients treated with S-ketamine will have a lower risk of POD. We have reason to believe that S-ketamine can significantly reduce postoperative pain scores and opioid consumption, which would be consistent with our previous findings (Zhu et al., 2023). We have reviewed the literature to identify potential preoperative confounding variables, including age, ASA class, education level, comorbidities, electrolyte disturbances, malnutrition, smoking and drinking, history of falls, visual and auditory disorders, depression, dementia, functional status, frailty, and sleep disturbances. Potential confounding variables may bias the degree of association between the intervention and the primary outcome. It is therefore crucial to identify potential confounding variables. To our knowledge, this study is the first pragmatic study of S-ketamine.

High-quality evidence supports the intraoperative use of ketamine during THA and TKA to reduce postoperative pain and opioid consumption and reduce the incidence of postoperative nausea and vomiting (Riddell et al., 2019; Hannon et al., 2023). Ketamine was not found to significantly increase the risk of psychiatric side effects, such as hallucinations, nightmares, and delirium, compared with controls (Xu et al., 2019; Wang et al., 2020). Ketamine, as an adjuvant in orthopedic surgery, appears to be safe and effective. However, the meta-analysis by Hannon et al. (2023) recommends that ketamine be used with caution in elderly patients who are at risk for POD after surgery. The American Society for Enhanced Recovery and the Perioperative Quality Initiative Joint Consensus Statement on Postoperative Delirium Prevention states that subanesthetic doses of ketamine can cause psychosis and increase the risk of POD, and recommend that efforts be made to reduce the exposure of high-risk populations to ketamine (Hughes et al., 2020). S-ketamine, the dextrorotatory isomer of ketamine, has a similar pharmacological mechanism to ketamine but is more potent and has fewer psychotomimetic side effects (Trimmel et al., 2018). S-ketamine has multiple targets. The primary mechanism of its anesthetic and analgesic effects is the non-competitive antagonism of NMDA receptors. S-ketamine can also act on opioid receptors, cholinergic receptors, and GABA receptors, activate D_2_ dopamine receptors and L-type voltage-gated calcium channels, and block sodium channels and hyperpolarization-activated cyclic nucleotide-gated potassium channels. The above non-NMDA pathways also play an essential role in analgesia and anesthesia (Trimmel et al., 2018). S-ketamine is water- and lipid-soluble. After injection, it is widely distributed throughout the body and rapidly crosses the BBB. A maximum drug plasma concentration is reached 1–2 min after intravenous injection. However, no randomized controlled studies have evaluated the ability of S-ketamine to prevent POD.

There are significant differences between this study protocol and previous works that have evaluated the ability of ketamine to prevent POD, many of which can be considered to be the advantages of our trial. (1) The study interventions described in this protocol will be performed on patients undergoing intraspinal anesthesia. Although large, multicenter, randomized controlled studies have shown no significant differences in the effects of regional and general anesthesia on POD (Li et al., 2022), patients undergoing intraspinal anesthesia are not as significantly exposed to risky drugs such as sevoflurane, propofol, and benzodiazepines than patients who undergo general anesthesia. These drugs have been shown to negatively interfere with the neuroprotective effects of ketamine, which may be the reason for the failure of prior randomized controlled studies. (2) There is a shortage of randomized controlled studies on S-ketamine for POD prevention, and our trial will help to fill a gap in this area. (3) The strict inclusion and exclusion criteria of randomized controlled trials limit the external validity of test results. This is a pragmatic study, and as such patients with preoperative dementia, depression, and multiple comorbidities will be included. This will facilitate the external validity of trial conclusions.

## Ethics statement

The studies involving humans were approved by the Medical Ethics Committee of The Affiliated Hospital of Qingdao University. The studies were conducted in accordance with the local legislation and institutional requirements. The participants provided their written informed consent to participate in this study.

## Author contributions

YOZ: Data curation, Investigation, Methodology, Validation, Writing – original draft, Writing – review & editing. WF: Writing – original draft, Writing – review & editing. QK: Writing – review & editing. FS: Writing – review & editing. ZL: Writing – review & editing. WX: Writing – review & editing. QL: Writing – review & editing. YH: Writing – review & editing. XW: Writing – review & editing. CJ: Writing – review & editing. JG: Writing – review & editing. YAZ: Funding acquisition, Investigation, Supervision, Validation, Writing – review & editing.

## Footnotes

^1^ www.medsta.cn/software
